# Impact of the COVID-19 pandemic on breastfeeding in Israel: a cross- sectional, observational survey

**DOI:** 10.1186/s13006-022-00505-5

**Published:** 2022-08-26

**Authors:** Moran Blaychfeld Magnazi, Gaya Sartena, Michal Goldberg, Deena Zimmerman, Einat Ophir, Ravit Baruch, Rebecca Goldsmith, Ronit Endevelt

**Affiliations:** 1 Nutrition Division, Ministry of Health, Jerusalem, Israel; 2 School of Public Health, University of Haifa, Haifa, Israel; 3 Administration for Strategic and Economic Planning, Ministry of Health, Jerusalem, Israel; 4 Public Health Department, Faculty of Medicine, Tel Aviv University, Tel Aviv, Israel; 5 Maternal Child and Adolescent, Ministry of Health, Jerusalem, Israel; 6 Ministry of Health, Public Health Services, Tel Aviv, Israel

**Keywords:** Breastfeeding, Lactation, Lockdowns, COVID-19, Newborns, Mothers

## Abstract

**Background:**

Since March 2020, the world has been coping with the COVID-19 pandemic. One group particularly affected were mothers of newborns. The Israeli government imposed three lockdowns, with the first from 14 March to 11 May 2020. It had the strictest rules, with effects among mothers including panic and stress. These mothers coped with new challenges as they were often without help from the extended family, could not meet lactation counsellors in person, and stayed longer on maternity leave.

**Methods:**

A cross-sectional, observational study collected data via an online anonymous survey in Israel. From 27 April 2020 to 11 May 2020, the survey was distributed through Facebook groups for breastfeeding mothers. It contained 32 multiple choice and 10 open questions. Multivariate logistic regression analysis, with adjustment for potential factors, was performed to determine the pandemic-related factors influencing breastfeeding, including the decision to breastfeed longer than planned.

**Results:**

Five hundred eighty women participated in the survey. Most mothers were over 30, (mean age 32.55), married with an academic degree (81.5%). 127 (22%) women reported changes in their lactation plans. 85 (15%) responded that due to the COVID -19 pandemic they extended their breastfeeding period and 42 (7%) reported shortening it. A significant relationship was found between this extension and returning to work later than expected adjusted OR = 2.38 95% CI 1.46,3.87). When asked to rank steps national health authorities should take to encourage breastfeeding, the highest agreement (96%) was with maternity leave extension. More than 90% believed that receiving breastfeeding counselling at home and/or in hospital will encourage breastfeeding.

**Conclusions:**

This study demonstrated that most women did not change their breastfeeding patterns because of the lockdown though some did experience difficulties. Some lengthened their breastfeeding period, as, due to the pandemic, they stayed home longer than expected. This finding should be considered for future emergency situations.

**Supplementary Information:**

The online version contains supplementary material available at 10.1186/s13006-022-00505-5.

## Background

Since the beginning of 2020, the world has been coping with a global pandemic of coronavirus disease (COVID-19) due to SARS-Cov2 virus [1]. This pandemic has led to lifestyle and behavior changes including social distancing, quarantine, and lockdowns [2]. In Israel, the government imposed the first lockdown on the country’s residents from the 14 March 2020 until 11 May 2020 [3]. Israeli citizens, other than essential workers, were instructed to stay within 100 m of their home and schools with the exception of special education.

One group in particular affected by lockdown measures were parents of infants, especially mothers. According to informal staff reports, in many maternity wards, mothers were sent home as early as possible (36 h) to minimize crowding. Other maternity facilities increased rooming-in to prevent exposure in nurseries. Staff shortages due to staff quarantine and moving staff to COVID-19 wards led to fewer nurses available for breastfeeding counseling.

Once at home, many in-person medical and breastfeeding support services were offered primarily online. Lockdown rules often limited the possibility of family support, meeting with friends and physical activity, all of which are likely to have emotional effects especially at this vulnerable time of life. Some women extended their maternity leave, either by choice or because they were laid off or put on unpaid leave. The unemployment rate in Israel rose from 8.3% in 2019 to a high record of 35% in April 2020 in the general population, and over 40% in the female population [4]. The fact that the maternity leave was extended de-facto for many women as compared to normal times, provided an opportunity for a "natural experiment" which allowed examination of the effect of maternity leave extension on breastfeeding.

It is essential to breastfeed infants, especially during a pandemic [5]. Breastfeeding provides appropriate nutrition, and breast milk contains anti-infective and anti-inflammatory factors that pass to the baby [6, 7]. In general, Israel has a high rate of breastfeeding initiation. Based on a study of a representative sample of the 2009 birth cohort, 90% of women-initiated breastfeeding in the hospital [8]. According to 2019 data from a nationwide database of maternal child clinics, representing 7% of the population, at one month 50% of women were exclusively breastfeeding and an additional 33% were partially breastfeeding. By 6 months, these numbers were 18% and 45% respectively [5].

Considering this reality, the goal of this study was to examine the effect of the COVID-19 pandemic on breastfeeding patterns among Israeli mothers of infants up to 6.5 months of age. We examined the factors associated with the decision to prolong breastfeeding longer than planned, due to the pandemic. We hypothesized that the lockdowns, and resultant extended maternity leave, would lead to prolonged periods of breastfeeding.

## Method

### Study design

A cross-sectional, observational study collected data via an online anonymous survey in Israel between 27 April, 2020, and 11 May, 2020 (the last day of the first lockdown in Israel). The study was approved by the Faculty Institutional Ethics committee of the University of Haifa. All respondents provided electronic informed consent prior to initiation of the survey.

The survey was in Hebrew and was translated to English (Supplementary file 1). A multidisciplinary team of experts in the field of breastfeeding constructed the survey tool, specifically designed for this study. The team included researchers from the Ministry of Health Nutrition Division and the Maternal and Child Health Division. The questionnaire was pretested, in a pilot study, on a small representative group of mothers who breastfed during the lockdown period. The questionnaire was distributed as a Google form through social media (Facebook groups for breastfeeding mothers in Israel). Participants were encouraged to assist in the distribution of the survey link to expand the exposure. No incentives were offered.

### Study population

Women who were members of Facebook groups for breastfeeding mothers were invited to participate. The survey was publicized via the Facebook page of some of the researchers and was directed to Hebrew-speaking mothers.

We included in the analysis mothers of single newborns up to 6.5 months old who breastfed during the COVID-19 period. Participation was voluntary. Exclusion criteria were: mothers of babies older than 6.5 months, mothers who had stopped breastfeeding before the study period or had not breastfed at all, mothers with non-singleton births (twins/triplets etc.). Incomplete surveys were not included in the analyses.

### Data collection

The survey contained 32 multiple choice and 10 open questions regarding demographic characteristics (maternal age, marital status, number of children in the family), information regarding the type of birth, infant feeding practices before and during the COVID-19 pandemic, social support regarding breastfeeding, the employment status of the mother and her partner, and an open-ended question about the actions that can be taken to promote breastfeeding during this period. In this (open-ended) question, mothers were asked to rank the importance of each action to breastfeeding encouragement (encouraged /did not encourage / irrelevant). Also, participants were asked to describe the effect of the COVID-19 pandemic on their planned breastfeeding duration. Most questions were mandatory.

### Statistical analysis

All statistical analyses were performed using R software version 3.6.3 (R core team, distributed CRAN, February 2020).

Continuous variables are presented as means ± standard deviation (SD) and nominal variables as proportions. Multivariate logistics regression analysis was performed to determine the factors associated with the decision to prolong breastfeeding, i.e. due to the pandemic, breastfed longer than planned, with adjustment to potential confounders. The content analysis approach was used for data analysis. The narratives were analyzed to determine the key themes. The analysis involved indexing the data, sorting and selecting quotes for placing them in the appropriate thematic category and developing a final interpretation.

## Results

**Fig. 1:**
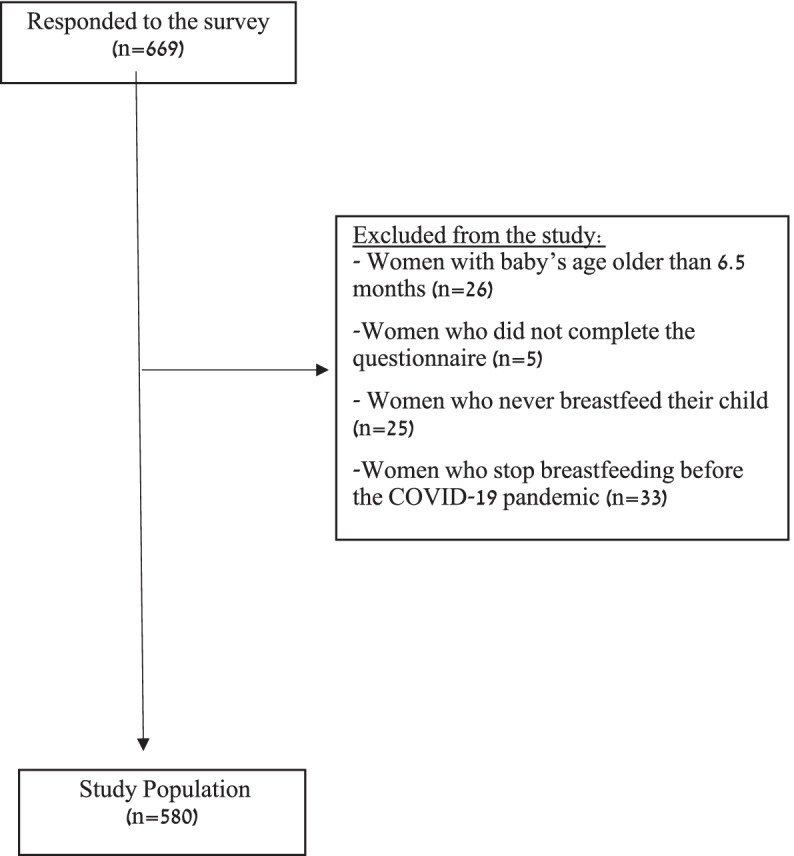
Study population flow diagram

Six hundred and sixty-nine women responded to the survey, of which 580 women were included in the analysis (Fig. 1). All closed questions required a response, whereas the open questions were not mandatory' There were very few incomplete questionnaires, and these were not included in the analyses. Characteristics of the study population are presented in Table 1. Participant’s mean age was 32.5 ± 4.2, and mean infant age was 3.04 ± 1.7 months. The majority of participants were multiparous (65%) and were still on maternity leave at the time of the survey.

**Table 1:** Characteristics of the study population

Characteristic	Category
Mother’s age (years, mean ± SD)	32.55 ± 4.2
Baby’s age (months, mean ± SD)	3.04 ± 1.7
Parity (n, %)	
Primiparous	203 (35)
Multiparous	377 (65)
Marital status (n, %)	
Married	572 (98.6)
Not married	8 (1.4)
Type of birth (n, %)	
Vaginal birth	497 (85.6)
Caesarean	83 (14.3)
Mothers employment status (n, %)	
Employed	70 (12)
Maternity leave	324 (56)
Unemployed	186 (32)
Partner employment status (n, %)	
Employed	457 (79)
Unemployed	115 (20)
Do not have a partner	8 (1)
Education level (n, %)	
No academic education	66 (11.3)
Academic education ^a^	514 (88.5)
Returned to work later than expected (%)	151 (26.0)

Abbreviations: *SD*, Standard deviation

^a^ Academic education level was defined as a first degree or higher

An analysis was carried out to assess correlation between rooming in following birth, and the length of breastfeeding. There was no statistical significance (*p* = 0.687).

One hundred and twenty-seven participants (22% of the study population) reported changes in their breastfeeding plans; 85 (15%) reported the COVID-19 pandemic prolonged their period of breastfeeding and 42 (7%) reported it shortened it.

The respondents who extended the breastfeeding period could cite one or more reasons for this extension. The reasons included belief that breastfeeding would protect again infection (70%), increased availability of the mother (66%), the breastfeeding relaxed the mother (33%), and for economic reasons (14%). Among those who shortened the breastfeeding period, 65% said it was due to lack of time because of other children at home, 34% cited stress and fear, 27% cited mood changes and 7% because of non-availability of breastfeeding counselling.

Participants’ report of support from friends, family, and/or lactation consultants since they gave birth, is depicted in Table 2. Most of the women had not been in quarantine due to COVID-19 disease or exposure to a confirmed patient since they gave birth. Of the 51 participants who were infected or exposed to a confirmed patient, only 4 reported separations from their baby (< 1% of the study population). Most women reported support from their partner, mother, mother-in-law, and friends. At the time of survey completion, 74% of the children were breastfed. Women reported lactation counselling during their hospital stay (67.8%), or after discharge (45.0%). Only 37.2% of mothers received both lactation counselling at the hospital and after discharge.

**Table 2:** Information about breastfeeding counselling during the first lockdown in Israel (*N* = 580)

Data	Proportion of participants (%)
Baby was born after the beginning of the lockdown	51 (9)
Separated from the baby	4 (0.7)
**In the hospital**	
** Full rooming in**	364 (64%)
** Partial rooming in**	88 (15%)
** Separated at night**	96 (17%)
** Baby in Premature infants nursery**	32 (5.5%(
**Breastfeeding intentions (prior to birth)**	
Breastfeed only	463 ( 80%)
Breastfeed and formula	104 (17.9%)
Only infant formula	8 (1.4%)
Undecided	5 (1%)
Breastfeeding counselling in the hospital	393 (67.8)
Breastfeeding counselling at home	261 (45)
Breastfeeding counselling in the hospital and at home	216 (37.2)
No. of breastfed (inc. expressed breast milk) during the study period of which	521 (90%)
-breastfed only	385 (74%)
- combined breastfeeding and formula	83 (16%)

Participants were given the opportunity to provide free-text answers regarding breastfeeding during the COVID-19 pandemic. Out of 580 participants, 96 women answered this question and discussed challenges they faced during the first lockdown. The main issues raised were lack of spare time due to other children in the house, financial difficulty, mental stress, and dissatisfaction with the lactation counselling which they had received (Table 3).

**Table 3:** Main themes expressed

Main themes	No. of responses	Quotes
Economic difficulties	32	“As a single mother of two, I am mainly worried financially” “I would be happy to stay at home with the baby until he was at a stage of eating more solid food, but, sadly, this is not financially possible. It is a shame that maternity leave is so short”
The need for lactation counselling	21	“Before giving birth, I really wanted to breastfeed, and I intended to breastfeed in the first few days, with the help of the lactation counselors but because of the Corona virus, the lactation counselors did not provide a service in the hospital where I gave birth, and this harmed the correct feeding process for my daughter” “I express milk, and feed with a bottle because of the crisis. I intended to breastfeed but because limited counselling in the hospital and lack of external guidance at the time of the crisis, and difficulty in feeding, it did not succeed”
Lack of availability because other children are at home	12	“It is hard to breastfeed for a long time, with other children at home, (I have 6-year-olds twins) and so when they were at home, I gave more formula than I would have given had they not been at home” “The main problem with breastfeeding during this time was that the mother hasn’t got time to rest as she is caring for the other children, and so she hasn’t enough milk” “The Corona stole my time with my baby as I was all the time with the older sister (2.8 years old). I wanted a corrective (restorative) experience following the first maternity leave “
Emotional stress because of the crisis	11	“I think that at the beginning of the crisis, the amount of milk decreased because of stress, but with the help of expressing and perseverance, it has returned to the regular amount” “As a result of the crisis less energy was available to invest in breastfeeding when there was a difficulty with the feeding”
Positive feelings	10	“I am definitely happy that I am exclusively feeding my baby at this time- this strengthens my feeling that I am protecting her. Incidentally, good quality lactation counselling in hospital is critical, especially for premature babies” “It actually worked out well for me. I went back for 3 weeks, and then there was the lockdown. So, I received more quality time with my daughter”
Other	10	“Breastfeeding is very important especially when there is a danger to health- the transfer of antibodies from the mother to the baby. It should be encouraged in any way” “In my opinion, it is extremely important to encourage breastfeeding, with or without connection to this crisis or others”

Table 4 shows the results of a multivariate logistic regression analysis of factors associated with longer than planned breastfeeding due to the pandemic. We adjusted for all demographic variables, namely mother’s age, baby’s age and education level. Marital status was left out because of the lack of variation in this variable. Mother's age was analyzed as a continuous variable, while the baby's age was analyzed as a binary variable, with two categories: below age 3.5 months and above. The cutoff age of 3.5 months was chosen as this is the duration of the paid maternity leave in Israel. Returning to work later than expected (vs. as expected) was significantly associated with prolonged breastfeeding, with those mothers being 2.38 (95% CI 1.46,3.87) times more likely to breastfeed for a longer period than expected.

**Table 4:** Multivariate analysis associated factors with prolonged^a^ breastfeeding due to the pandemic

Criterion	Adjusted Odds ratio	95% Confidence interval
Baby’s age (≥ 3.5 months vs. < 3.5 months)	1.46	(0.91,2.36)
Baby is the only child (vs. siblings)	1.19	(0.71,1.94)
Returned to work later than expected (vs. returned to work as expected)	2.38	(1.46,3.87)
Mother's age	1.00	(0.98,1.00)
Academic education vs. no academic education	2.01	(0.89,5.45)

^a^- longer than initially planned

Table 5: Present Odds Ratio (OR) between two comparison groups. The reference group is mentioned in the table. OR's are adjusted to mothers' age and to one another.

^***^p < 0.001

### Actions to encourage breastfeeding

In response to which factors could help in encouraging breastfeeding, mothers could answer either based on their current experiences or on what they consider could help, or a combination (experience and ideas regarding future desirable actions). High proportions of the study participants reported breastfeeding encouragement in prolonged maternity leave (96.0%), having the possibility to work from home (86.0%), receiving lactation counselling at home (93.5%) and/or in the hospital (92%), and in watching training videos on breastfeeding (81.0%). However, only 72.5% of the participants believed virtual meeting groups with other mothers would encourage breastfeeding (Table 5).

**Table 5:** Mothers' ranking of actions for breastfeeding encouragement (based on mothers' experience or ideas or both)

Factors	Responses N, (%)
Prolonged maternity leave	555 (96)
Lactation counselling at home	541 (93.5)
Lactation counselling at the hospital	532 (92)
Expressing instruction	516 (90)
Possibility to work from home	500 (86)
Telephone lactation counselling	472 (81.5)
Consult videos for breastfeeding	468 (81)
Virtual group meeting with other mothers	421 (72.5(

## Discussion

This study examined the association between of a tight nationwide lockdown due to the COVID-19 pandemic, and mother’s breastfeeding experiences with their newborn, including any impact on length of breastfeeding period. Only 22% of the women reported that the COVID-19 pandemic influenced their plans to breastfeed, with most prolonging the breastfeeding period. Prolonged maternity leave due to the pandemic was significantly associated with prolonged breastfeeding. This result may shed light on the effect of a policy change, namely extending the paid maternity leave, on breastfeeding encouragement. This finding is in concert with a study from Belgium [9] which found that 91% of the breastfeeding mothers claimed they did not change their infant feeding due to the COVID-19 pandemic [9]. The primary reasons given for prolonged breastfeeding were the prolonged maternity leave and a desire to protect the infant against the COVID-19 through breastmilk. The main reasons provided for a shortened period of breastfeeding were a reduction in milk production due to anxiety and increased childcare responsibilities at home [9].

A study conducted in the United Kingdom [10] (UK) among mothers of infants ≤ 12 months of age during the pandemic found that 41.8% of women prolonged their breastfeeding due to lockdown. However, 27.0% stopped breastfeeding before they were ready. The main reasons given for breastfeeding cessation were lack of face-to-face support, worries about the safety of breastfeeding and for a few, infection with COVID-19. Authors reported an association between a lower education level and ethnic background and breastfeeding cessation. These were not seen in our study, possibly due to our more homogenous sample of participants. Some of these difficulties were also raised in our study, namely inadequate family support, lack of in-person lactation counselling.

In our study we asked if lack of contact with their family or friends affected their breastfeeding, 16% of women reported a change. While some women claimed that lack of contact meant less influences not to breastfeed, others reported a negative effect of the lockdown on their mood and social support mechanisms. Depression during lockdown has been reported in other studies [11]. Social support has been previously described as an important contributor to a mother’s ability to breastfeed [12]. It is important to encourage breastfeeding especially during respiratory viruses because of the advantages of breastfeeding for the mother and the baby [13]. International health organizations recommend that mothers with suspected or confirmed COVID-19 should be encouraged to initiate or continue to breastfeed. The benefits of breastfeeding outweigh the potential risks or transmission [7, 14, 15].

Limitations of this study include a potential sampling bias. Since we used social media to recruit participants, we used a convenience sampling method. Our study was sent as a link only in one social media- Facebook. Hence, the study population includes only women who had an internet connection and were actively using this social media. Furthermore, the incidences of higher university degrees are higher than in the general population. According to the Central Bureau of Statistics (Israel), in 2020, 32% of women age 15 and over, have an academic degree. As such, the results may not be representative of the general population.

## Conclusion

The findings of this study demonstrate that for most of the women the pandemic did not influence breastfeeding duration. For others, the period was lengthened and for a small minority, the breastfeeding period was shortened. The study also showed that some women experienced difficulties with breastfeeding during the nationwide lockdown. Prolonged maternity leave was positively associated with prolonged breastfeeding, which is highly encouraged by all health organizations for various health-related concerns. The difficulties raised (lack of spare time due to other children in the house, financial difficulty, mental stress, and dissatisfaction with the lactation counselling which they had received) in maintaining breastfeeding raised in this population-based survey, should be taken into account for preparing for future emergency situations.

## Supplementary Information

**Additional file 1.** Topic - Breastfeeding questionnaire during the Corona pandemic.

## Data Availability

Not applicable.

## Abbreviations

COVID-19: Coronavirus disease
SD: Standard deviation
UK: United Kingdom
