# Supplementary material for: Impact of the COVID-19 pandemic on breastfeeding in Israel: a cross- sectional, observational survey
Source: Int Breastfeed J. 2022 Aug 26;17:61. doi: 10.1186/s13006-022-00505-5 (PMC9412797; doi:10.1186/s13006-022-00505-5)
Supplement: Supplementary file 1 — Additional file 1. Topic - Breastfeeding questionnaire during the Corona pandemic. [file 13006_2022_505_MOESM1_ESM.docx]

**Topic - Breastfeeding questionnaire during the Corona pandemic**

Opening:

Hello and congratulations,

This questionnaire is intended for mothers of healthy infants up to the age of six months (not twins)

Thank you for completing the following short questionnaire (less than 10 minutes).

The purpose of the questionnaire is to examine the impact of the corona crisis on your baby's feeding patterns.

The questionnaire was developed by the Nutrition Division and the Department of Mother and Child at the Ministry of Health, the results of which will serve the Ministry of Health in examining changes in care / guidance and policies regarding infant nutrition in emergencies and in the future.

For any questions, please contact Dr. Moran Blaychfeld Magnazi at moran.magnazi@moh.gov.il

The questionnaire is anonymous and we ask to fill it out only once.

By filling out this questionnaire, I express my consent to participate in this survey - yes / no

1. What is your age? _______ years

2. Marital status: living in a relationship / living alone

3. How many children in the house, including the baby? ________

4. Date of birth of the baby:________________

5. Age of the baby in months? _________

6. Type of birth? Normal, elective caesarean, emergency caesarean, other

7. During the stay in the maternity ward the baby was in? A. Full rooming-in / zero separation b. Partial rooming-in c. Nursery for the night d. Prematurity ward e. Other

8. Are you in isolation or have you been in isolation since the baby was born? Yes/ No

9. Did you have to be apart from your baby due to the corona outbreak? A. I was not apart from the baby due to Corona b. Yes for one day c. Yes, for a few days, less than a week d. Yes, more than a week

10. Do you go out with your baby to get some fresh air? Yes/No

11. What best describes your baby's diet these days: a. Breast milk only (breastfeeding or expressing) b. Breast milk combined with formula (infant formula) c. Formula only d. Breast milk and some solid foods e. Formula and some solid food f. Other

12. What best describes your baby's diet before the outbreak of the corona crisis: a. Breast milk only (breastfeeding or expressing) b. Breast milk combined with formula (infant formula) c. Formula only d. Not relevant - I gave birth during the crisis e. Other

13. How did you plan to feed the baby before giving birth? A. Breast milk only (breastfeeding or expressing) b. Breast milk combined with formula (infant formula) c. Formula only d. Other

14. If there is a change between the plan and what is happening now - to what extent do you think the corona crisis affected the change? Did not affect at all / affected a little / affected very much

15. Did you seek help from a breastfeeding counselor at the hospital? Yes/ No

16. Did you get help from a breastfeeding counselor after you got home? Yes No

17. How long has you been breastfeeding or giving expressed milk to your baby? A. Still breastfeeding / expressing b. I did not breastfeed / express at all c. I stopped after a few days after giving birth d. I stopped after a week to a month e. I stopped after a month to two months f. I stopped at two months to 3 months g. I stopped after 3 months

18. Up to what age in the months do you plan to breastfeed / express breast milk for the baby? _____ months

19. Due to the crisis, did you decide to breastfeed / express for your baby for longer? Yes / No / Not relevant

20. If you knew there would be a corona epidemic would you in retrospect consider giving a different diet? Yes / no / do not know

21. Why did you decide to extend the duration of breastfeeding / expressing? (You can mark more than one answer) a. More availability and time at home b. I realized that breastfeeding protects against infections c. Cheaper d. Soothing e. Other

22. Does the crisis make you less willing to express/ breastfeed the baby? Yes / No / Not relevant

23. If so, why are you breastfeeding less/ expressing less? (You can mark more than one answer) a. Stress / anxiety b. Lack of availability due to additional children currently at home c. Concern for older parents and family d. Fear of infecting the baby e. Decreased mood f. Other

24. Do you receive / have you received social support to encourage breastfeeding (more than one answer can be marked) from my spouse / mother-in-law / my mother / friends / do not receive / received support / irrelevant / other

25. Does the lack of contact with friends / family affect breastfeeding? Yes / No / Not relevant

26. How does the lack of contact with friends and family affect breastfeeding? _____________________________________________________________________________________________________________________________________________________________

We want to hear your opinion, in order to help us encourage breastfeeding in future emergencies.

Rate the impact level in your opinion of the following actions according to your experience

1. Very helpful 2- Will help 3- Will not help 4- Not relevant to me

27. Obtaining breastfeeding counseling at the hospital

1. Receiving breastfeeding counseling at home
2. If not provided frontally, obtain breastfeeding advice by telephone
3. Watch breastfeeding instructional videos
4. Training for expressing milk
5. Virtual group meeting with additional mothers
6. Possibility to work from home
7. Extension of maternity leave
8. Before the birth and before the onset of the corona crisis what was your employment situation like?

a. I did not work before the birth

b. I was self-employed

c. I worked as an employee

d. I worked as an employee and was self-employed

e. Other

1. What is your current employment situation during the crisis?

a. I'm still on maternity leave for 14 weeks

b. I'm on unpaid leave

c. Works full-time as usual outside the home

d. Works full time but from home

e. Works but fewer hours

f. I'm on paid leave

g. I was fired from my job

h. Other

37. What is your spouse's current employment situation?

a. Works normally outside the home

b. Works as usual from home as well

c. Works at lower hours

d. On unpaid leave

e. On paid leave

f. Irrelevant- I do not have a spouse

g. Other

38. At what age of the baby in months did you plan to return to work assuming there was no corona crisis? ______ months

39. Has the time to return to work changed due to the crisis? a.Your return time was brought forward. b. Your return time was delayed c. The time of your return to work has not changed d. Do not know e. Other

40. What will be the baby's age in months, when you will return to full or part-time work? _________

41. Finally, what is your highest certificate or degree?

a. Less than 12 years of schooling

b. A high school diploma

c. Professional certificate

d. First degree

e. Master's degree or higher

42. If you are interested in us contacting you later in addition to mailings, and to answer another questionnaire, please write your email address:_______________________________________

43. If you have anything to add we would love to hear your opinion

Thank you very much, we wish you and your family good health.
